# Roasted *Astragalus membranaceus* Inhibits Aβ25–35-Induced Oxidative Stress in Neuronal Cells by Activating the Nrf2/HO-1 and AKT/CREB/BDNF Pathways

**DOI:** 10.3390/antiox13111311

**Published:** 2024-10-28

**Authors:** Yun-Jeong Ji, Min Hye Kang, Sin Hee Han, Geum-Soog Kim, Hyung Don Kim, Gwi Yeong Jang

**Affiliations:** 1 Department of Herbal Crop Research, National Institute of Horticultural and Herbal Science, Rural Development Administration, Eumseong 27709, Republic of Korea; jyj2842@korea.kr (Y.-J.J.); mohak2@korea.kr (M.H.K.); herbman@korea.kr (S.H.H.); kimgs0725@korea.kr (G.-S.K.); 2 Department of Biochemistry, School of Life Sciences, Chungbuk National University, Cheongju 28644, Republic of Korea

**Keywords:** *Astragalus membranaceus*, Alzheimer’s disease, BDNF pathways, hippocampal neuronal cells, oxidative stress

## Abstract

(1) Background: *Astragalus membranaceus* (AM) has antioxidant and anti-inflammatory effects, but its specific mechanism of action in the brain is still unclear. In this study, we developed a roasting process to maximize the cognitive improvement impact of AM. We focused on enhancing physiological activity to enhance the brain neuron protection effect and alleviate neuronal damage caused by neurodegenerative diseases. (2) Methods: AM was roasted at 260 °C for 20, 30, or 40 min, and the hot water extracts were tested on HT22 cells for ROS levels, apoptosis, and antioxidant protein expression. The effect on the BDNF-AKT-CREB pathway under stress was also analyzed. (3) Results: Roasted AM decreased ROS production and the expression of apoptosis-related factors while activating the expression of antioxidant proteins in HT22 cells treated with Aβ25–35. In particular, 30 min roasting (R-AM2) significantly reduced ROS production, inhibited cell death, and increased antioxidant protein expression. The Nrf2 pathway was activated Bax, and cleaved caspase-3 levels were reduced. BDNF and p-CREB expression were increased by 20% and 50–70%, respectively. In the MAPK pathway, p-ERK levels were increased by 30%, and p-P38 levels were increased by approximately 20%. (4) Conclusions: These findings suggest that roasted AM upregulates brain-derived neurotrophic factor (BDNF) in HT22 cells, providing neuroprotective effects by activating the AKT/CREB/BDNF pathway and inhibiting neuronal apoptosis. Therefore, roasted AM shows potential as a neuroprotective agent for preventing or treating neurodegenerative diseases, such as Alzheimer’s, linked to BDNF deficiency.

## 1. Introduction

Alzheimer’s disease (AD) is a neurodegenerative disease in which memory and cognitive function progressively decline, and the leading causes are the accumulation of beta-amyloid (Aβ) protein and hyperphosphorylation of tau protein. This process causes damage to neurons and, over time, leads to an overall decline in brain function [1]. Aβ accumulation begins with the degradation of amyloid precursor protein (APP), and the Aβ produced here is converted into monomers, oligomers, polymers, and insoluble plaque forms. Of these, Aβ oligomers are particularly toxic to neurons and are known to be the primary pathological factor promoting the progression of AD [2,3].

Promising approaches to preventing or slowing AD progression include modulating critical cellular pathways. Brain-derived neurotrophic factor (BDNF) and mitogen-activated protein kinase (MAPK) signaling pathways have been attracting attention as therapeutic targets for AD [4]. BDNF plays an important role in promoting neuronal survival and growth. BDNF binds to the TrkB receptor and activates the PI3K/Akt and MAPK/ERK pathways to promote neuronal survival and suppress damage. In addition, BDNF plays an important role in neuroprotection by reducing Aβ toxicity in Alzheimer’s disease (AD) [5,6]. However, in the chronic pathological environment of AD, excessive activation of the MAPK pathway increases the production of inflammatory cytokines, inducing neuronal cell death and worsening the pathological condition of AD [7,8]. Therefore, inhibition of the MAPK pathway can be expected to have a neuroprotective effect by reducing the neuronal damage caused by oxidative stress. Currently, one of the main approaches to treating Alzheimer’s disease (AD) is to reduce Aβ accumulation. However, many clinical trials have reported that therapies targeting Aβ have failed to show the expected improvement in cognitive function [9]. This suggests multiple pathways, such as enhancing BDNF signaling and inhibiting the MAPK pathway, are needed for AD treatment [10]. Against this backdrop, natural product-based treatments are attracting attention [11,12]. In particular, considering the limitations of synthetic drugs and the complexity of AD pathology, natural products can be evaluated as more effective treatment alternatives due to their unique chemical structures and novel mechanisms of action.

*Astragalus membranaceus* (AM) is known as a medicinal herb with various effects in traditional medicine, such as anti-inflammatory [13], antioxidant, anti-diabetic [14], anti-cancer [15], immunomodulatory [16], anti-aging, and whitening effects [17,18]. Recent studies have demonstrated potential therapeutic effects on neurodegenerative diseases such as AD, and isoflavones, one of the main components of AM, reduce Aβ accumulation and suppress oxidative stress and inflammation [19]. In particular, isoflavones such as calycosin and formononetin are known to lower inflammatory cytokine (IL-1β, TNF-α) levels and improve cognitive function decline by reducing Aβ accumulated in the hippocampus in AD models [19,20].

These natural ingredients are becoming important research subjects for AD treatment. However, natural bioactive substances are sensitive to environmental factors and are readily decomposed or have low absorption rates due to light, oxygen, and temperature [21]. To solve these problems, processing is necessary, and among them, roasting is used as a method to increase the bioavailability of the physiologically active components of natural products and to enhance the pharmacological effects [22].

Roasting deepens flavor and texture, improves storage properties, and prevents oxidation of foods [23]. Roasting can break down plant cell walls to release active ingredients such as isoflavones and saponins, and enhance antioxidative and anti-inflammatory effects. For example, roasting ginger produces potent anti-inflammatory and antioxidant ingredients such as 6-shogaol and zingerone, and roasting licorice can induce the production of reactive oxygen species (ROS) [24], inhibit cell death, and reduce damage caused by oxidative stress [25]. Roasting also increases the concentration of saponins and isoflavones in AM, which reduces oxidative stress and improves immune function. Our previous study showed that ginseng saponins also showed an enhanced neuroprotective effect when heat-treated, as the content of specific ginsenosides (e.g., Rg3) increased. This heat treatment induces structural changes in saponins, which allows them to exert more potent neuroprotective effects [26]. Astragaloside IV extracted from AM contributes to neuroprotection by regulating the NF-κB, PI3K, Akt, IκB, and MAPK signaling pathways, suggesting therapeutic potential in neurodegenerative diseases such as AD [19,27].

In this study, we optimized roasting time and temperature to enhance AM’s physiological activity. AM prevented neuronal cell death by enhancing BDNF signaling and inhibiting MAPK signaling in HT22 cells treated with Aβ. We aimed to provide a foundation for new approaches to the prevention and treatment of neurodegenerative diseases.

## 2. Materials and Methods

### 2.1. Preparation and Extraction of Roasting Samples

AM roots were harvested in Jecheon, Chungcheongbuk-do, in 2022. Dried AM root was cut into 3–4.5-mm pieces, and 15 kg of AM were roasted in a rotary roaster (Taepyung Co., Ltd., Jincheon-gun, Chungcheongbuk-do, Republic of Korea) at 260 °C for 20 min (1R-AM), 30 min (2R-AM), or 40 min (3R-AM). To prepare the dried and roasted AM extracts (NR-AM, RAMs), 300 g of dried and roasted AM were extracted twice with 2.4 L of water at 98 ± 2 °C for 7 h. The extracts were filtered and lyophilized for analysis. The NR-AM and RAM samples were dissolved in dimethyl sulfoxide (DMSO; Sigma-Aldrich, St. Louis, MO, USA) and stored at −80 °C.

### 2.2. Analysis of Roasted AM Water Extracts by HPLC-UVD

NR-AM and RAMs (0.5 g) were dissolved in 50 mL of 70% ethanol, shaken, and passed through a 0.2-μm membrane filter (Pall Co., Ann Arbor, MI, USA). The isoflavone composition was analyzed by high-performance liquid chromatography with UV-visible detection (HPLC system: 1200 series, Agilent Technologies, Santa Clara, CA, USA; column: Triart C18, 3 µm, 100 × 4.6 mm, YMC Co., Kyoto, Japan). The mobile phase consisted of 0.1% formic acid in acetonitrile (A) and 0.1% formic acid in water. The gradient was as follows: 0 min (0% A), 0–5 min (0–0% A), 5–7 min (0–10% A), 7–12 min (10–20% A), 12–16 min (20–26% A), 16–17 min (26–26% A), 17–22 min (26–33% A), 22–32 min (33–60% A), 32–33 min (60–95% A), and 33–41 min (95–95% A). The flow rate, detection wavelength, injection volume, and column temperature were set at 1.0 mL/min, 250 nm, 10 µL, and 30 °C, respectively. The isoflavone standards—calycosin 7-glucoside, ononin, calycosin, and formononetin—were purchased from Sigma-Aldrich. HPLC-grade water and acetonitrile (ACN) were purchased from Fisher Scientific (Pittsburgh, PA, USA); all other chemicals were of reagent grade.

### 2.3. Cell Culture

Dulbecco’s modified Eagle’s medium (DMEM), phosphate-buffered saline, penicillin/streptomycin, trypsin, and fetal bovine serum (FBS) for cell culture were purchased from Gibco (Waltham, MA, USA). HT22 hippocampal neuronal cells (Merck KGaA, Darmstadt, Germany) were cultured in DMEM supplemented with 10% FBS and 1% penicillin–streptomycin at 37 °C in a 5% CO₂ incubator. The medium was replaced every 2 days for subculture. Cells from passages 5–10 were used in the experiments.

### 2.4. Cell Viability Assay

MTS (3-(4,5-dimethylthiazol-2-yl)-5-(3-carboxymethoxyphenyl)-2-(4-sulfophenyl)-2H-tetrazolium (Promega, Madison, WI, USA) DMSO, and the antibody used to assay cytotoxicity were purchased from Sigma-Aldrich; all other chemicals were of reagent grade. Cell viability was assessed using the MTS assay. HT22 cells were seeded at a density of 1.0 × 10⁴/well in 96-well microplates and incubated for 24 h. After culturing for 24 h, the medium was removed, and the cells were pretreated with RAM1, RAM2, and RAM3 for 1 h. Afterwards, 10 μM Aβ25–35 peptide (Bachem AG, Bubendorf, Switzerland) was added to each group for 24 h. After the incubation was completed, the medium was removed, treated with MTS reagent (Promega) for 1 h, and absorbance was measured at 490 nm using a multiplate reader (BioTek, Winooski, VT, USA).

### 2.5. Intracellular ROS Generation

Dichloro-dihydro-fluorescein diacetate (DCF-DA) fluorescent dye was used to measure intracellular ROS levels. HT22 cells were cultured in 96-well plates at 1.0 × 10⁴/well for 24 h, and the medium was replaced with the extract in a medium without FBS and then cultured for 24 h. After culture, the medium was removed, and 10 μM Aβ25–35 was treated for 30 min. After washing with PBS, 20 μM, DCF-DA was added to the culture medium and treated at 37 °C for 40 min. After removing the DCF-DA and washing with PBS, the degree of fluorescence (excitation 485 nm and emission 535 nm) was measured using a fluorescence reader (BioTek, Winooski, VT, USA).

### 2.6. Protein Extraction

HT22 cells were pretreated with 1R-AM, 2R-AM, 3R-AM (200 μg/mL), and Aβ (10 μM/mL) for 24 h. Whole HT22 cell lysates were prepared in RIPA buffer (GenDEPOT, Katy, TX, USA) containing a cocktail of protease and phosphatase inhibitors (GenDEPOT). Cell lysates containing equal amounts of total protein were quantified using the Bradford assay (Bio-Rad Laboratories, Hercules, CA, USA). Proteins were mixed with 4× loading buffer and boiled for 10 min.

### 2.7. Western Blotting

Cells were harvested 18 h after treatment with the extracts and Aβ and lysed on ice for 30 min in ice-cold RIPA buffer containing 10 mM Tris-HCl (pH 7.5), 0.1% NP-40, 0.5% sodium deoxycholate, 0.1% SDS, 1 mM sodium orthovanadate, 120 mM sodium chloride, 1 mM phenylmethylsulfonyl fluoride, 10 μg/mL leupeptin, and 1 μg/mL aprotinin. The lysates were centrifuged at 12,000 rpm for 10 min at 4 °C to remove cell debris and membrane components. The supernatant was collected, and protein concentrations were standardized using the Bradford method. Subsequently, 20 μg of total protein from each sample was separated by 8% or 10% sodium dodecyl sulfate-polyacrylamide gel electrophoresis (SDS-PAGE). The proteins were transferred onto polyvinylidene fluoride (PVDF) membranes (Bio-Rad Laboratories, Hercules, CA, USA). The membranes were blocked at room temperature for 1 h in a blocking buffer containing 3% bovine serum albumin (GenDEPOT) in TBS-T (50 mM Tris-HCl [pH 7.5], 150 mM NaCl, 0.1% Tween 20) to prevent nonspecific binding of antibodies. Western blotting was performed using rabbit anti-Nrf2 (1:1000), anti-HO-1 (1:500), anti-catalase (1:1000), anti-GPx (1:1000), anti-SOD2 (1:1000), anti-Bax (1:500), anti-Bcl-2 (1:500), anti-Bcl-xL (1:500), anti-cytochrome c (1:1000), anti-caspase-9 (1:1000), anti-caspase-3 (1:500), anti-p-Akt (1:500), anti-Akt (1:500), anti-p-CREB (1:500), anti-CREB (1:500), and anti-BDNF (1:500) primary antibodies (all from Cell Signaling Technology, Beverly, MA, USA). The membranes were incubated with the primary antibodies overnight at 4 °C, washed three times, and incubated for 2 h at room temperature with the mouse anti-β-actin secondary antibody (1:1000; Santa Cruz Biotechnology, Santa Cruz, CA, USA), which served as the loading control. Protein bands were detected using an Enhanced Chemiluminescence Western Blotting Detection Kit (Bio-Rad) and visualized with the ChemiDoc Imaging System (Bio-Rad). Relative protein expression levels were quantified using ImageJ Version 1.38 software (National Institutes of Health, Bethesda, MD, USA).

### 2.8. Experimental Data Analysis

The results are presented as means ± standard deviations (SDs) of three independent measurements. Statistical analysis was conducted using Prism 7.0 software (GraphPad Software, La Jolla, CA, USA). Non-linear regression analysis was performed to assess the relationships between groups, and corresponding curves were generated. The significance of the differences between groups was evaluated using independent *t*-tests and one-way analysis of variance (ANOVA), followed by post hoc tests. For pairwise comparisons, Tukey’s post hoc test or Fisher’s least significant difference (LSD) test were utilized. Effect sizes were assessed to clarify the magnitude of the observed differences. Additionally, 95% confidence intervals (CIs) for the mean differences and effect sizes were calculated to assess the precision and reliability of the estimates. The threshold for statistical significance was regarded as *p* < 0.05. During the revision of this paper, AI was used to improve grammar and readability but did not affect the scientific content and data analysis.

## 3. Results

### 3.1. HPLC Analysis of RAMs

**Figure 1:**
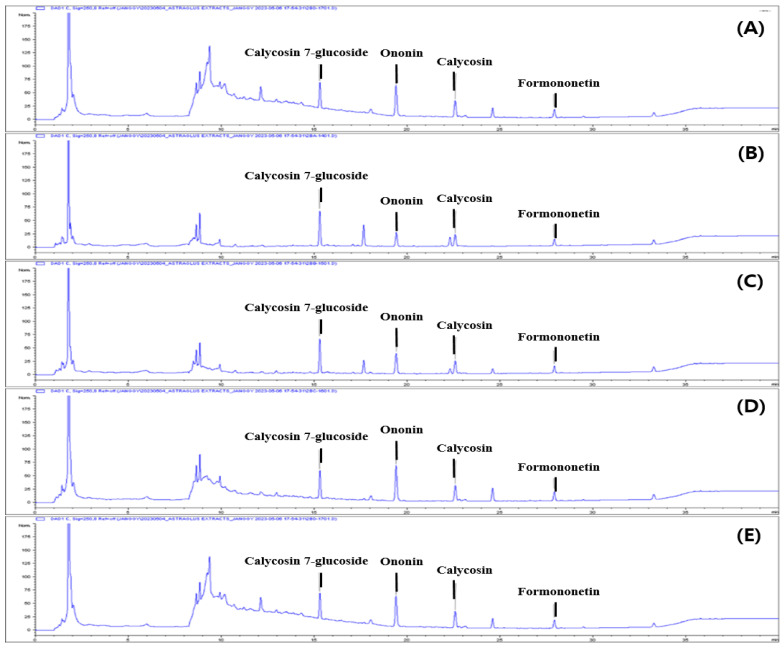
Typical chromatograms of AM water extracts according to roasting time. Samples: (**A**) Standards, (**B**) AM water extract, (**C**–**E**) Water extracts from AM roasted at 260 °C for 20, 30, and 40 min, respectively.

Much research has focused on the effect of roasting on bioactive substances. The effect of roasting on the quantities of AM, calycosin, formononetin, and their glycosides (calycosin-7-O-β-D-glucoside and ononin) was quantitatively analyzed. The contents of flavonoid compounds—calycosin, formononetin, and their glycosides—in the AM extracts were analyzed by HPLC (Figure 1).

### 3.2. Effect of Roasting on the Isoflavone Composition of AM

**Figure 2:**
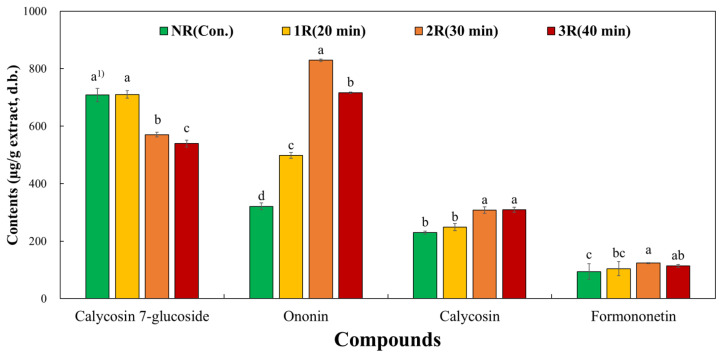
Isoflavone compositions of roasted AM water extracts according to roasting time. Isoflavone content according to roasting time. One-way ANOVA with Tukey’s post hoc multiple comparison test. Data are means ± standard errors of the mean of triplicate experiments. ^(1)^ Different letters on bars indicate a significant difference (*p* < 0.05) among samples with different roasting times.

The content of calycosin-7-O-β-D-glucoside, a glycoside of calycosin, was highest in NR-AM at 708.04 ± 23.47 μg/g, followed by 1R-AM at 709.88 ± 11.85 μg/g, 2R-AM at 570.43 ± 5.46 μg/g, and 3R-AM at 539.19 ± 27.01 μg/g. The content of ononin was highest in the 2R-AM extract at 828.87 ± 12.61 μg/g, whereas the 3R-AM extract roasted for 40 min had the lowest content at 716.30 ± 24.47 μg/g. The contents of calycosin and formononetin in the 2R-AM extract (calycosin: 307.69 ± 11.09 μg/g, formononetin: 123.56 ± 8.75 μg/g) were higher than those in the NR-AM extract (Table 1). The highest levels of calycosin and formononetin were observed in the 2R-AM extract. The content of calycosin-7-O-β-D-glucoside was highest in the NR-AM extract, whereas the ononin content was highest in the 2R-AM extract. The isoflavone composition results are shown in Figure 2.

### 3.3. Viability of Aβ-Treated HT22 Cells

**Figure 3:**
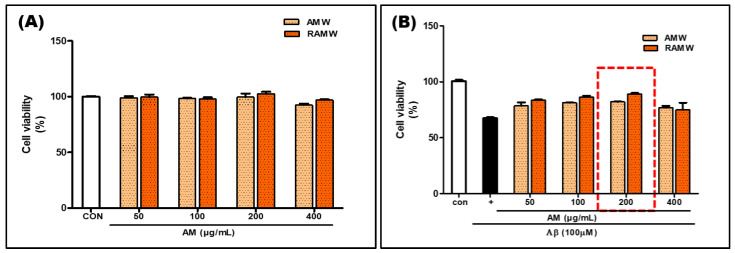

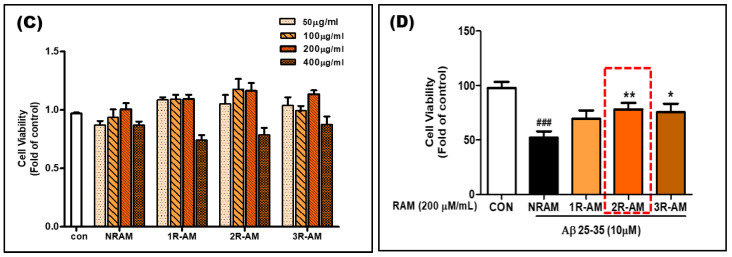
Viability of HT22 cells. (**A**,**B**) HT22 cells were treated with AM and RAM (50, 100, and 200 μg/mL) for 24 h to assess toxicity. (**C**) Effects of AM and RAM on cell viability after treatment with 10 μM Aβ for 24 h. (**D**) Cell viability after RAM treatment (200 μg/mL) and Aβ incubation for 24 h, assessed by MTS assay. The 2R-AM treatment group shows a higher cell viability than other concentrations, indicating a statistically significant difference. Control groups were treated with the same volume of conditioned medium (0.2% DMSO). Significance was determined by one-way ANOVA with Tukey’s post hoc multiple comparison test; ^###^ *p* < 0.001 compared with the control (white bar); * *p* < 0.05, ** *p* < 0.01 compared with Aβ25–35 treatments (black bar).

To evaluate the effects of raw AM and roasted AM on cell viability, HT22 cells were treated with the extracts at 50, 100, 200, and 400 μg/mL concentrations for 24 h and subjected to an MTS assay. The AM and roasted AM extracts at 400 μg/mL exhibited toxicity (Figure 3A). The roasted AM extract at 200 μg/mL resulted in the highest cell viability (Figure 3B). The roasted AM extract led to higher cell viability than raw AM. The extracts at 50–200 μg/mL were not toxic, but they showed toxicity at 400 μg/mL. At the optimal concentration of 200 μg/mL, 2R-AM showed the highest survival rate (49.7%) in Aβ-stimulated HT22 cells, indicating a considerable neuroprotective effect (Figure 3D).

### 3.4. Effect of RAM on ROS Generation in Aβ-Treated HT22 Cells

**Figure 4:**
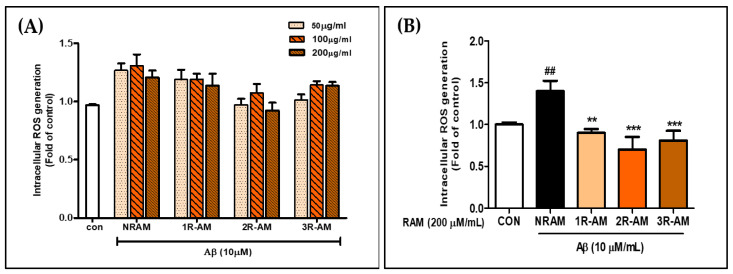
Inhibition of Aβ25–35-induced oxidative stress in HT22 cells by RAM. (**A**) ROS production in HT22 cells based on roasting time and RAM concentration. (**B**) Inhibition of ROS generation after treatment with RAM (50, 100, 200 μg/mL) for 24 h and stimulation with Aβ25–35 (10 μM) for 30 min. ROS generation was visualized using confocal fluorescence microscopy and measured after DCF-DA staining. Control groups were treated with the same volume of conditioned medium (0.2% DMSO). Significance was determined by one-way ANOVA with Tukey’s post hoc multiple comparison test; ^##^ *p* < 0.01 compared with the control (white bar); ** *p* < 0.01, *** *p* < 0.001 compared with Aβ25–35 treatments (black bar).

To evaluate the effect of R-AM on intracellular ROS levels according to roasting time, cells were treated with 50, 100, and 200 μg/mL extracts and subjected to DCFH-DA analysis. The 2R-AM (roasted for 30 min) extract showed the most excellent neuroprotective effect at all concentrations (Figure 4A). In Aβ-treated HT22 cells, ROS production increased 1.5-fold when treated with vehicle but decreased by 37.9%, 50.1%, and 42.3% with 1R-AM, 2R-AM, and 3R-AM, respectively (Figure 4B). The 2R-AM showed the most significant inhibition of ROS production, confirming its neuroprotective effect against Aβ-induced cell death.

### 3.5. Activation of the Nrf2 Pathway and Antioxidant Defenses

**Figure 5:**
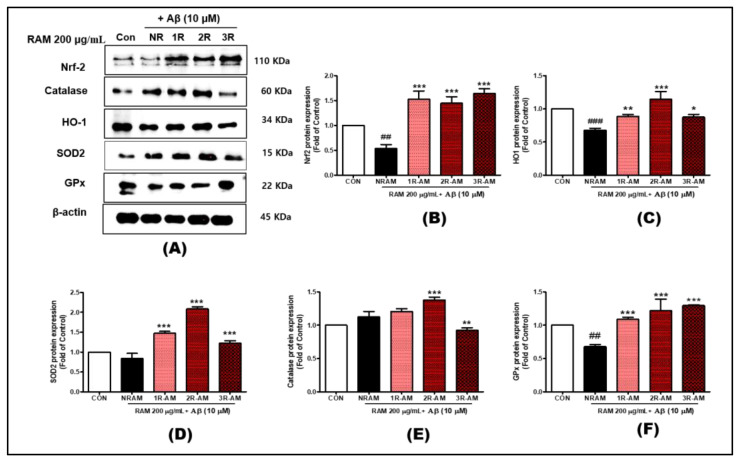
Representative Western blot image of Nrf2, HO-1, catalase, GPx, SOD2, and β-actin in HT22 cells. β-actin was used as the internal loading control. (**A**–**F**) Effect of RAM on the Nrf2, HO-1, catalase, GPx, and SOD2 protein levels in Aβ-treated HT22 cells. Control groups were treated with the same volume of conditioned medium (0.2% DMSO). Values are presented as standard deviations. Statistical analyses were conducted by one-way ANOVA followed by Fisher’s LSD test. ^##^ *p* < 0.01, ^###^ *p* < 0.001 versus the control group. * *p* < 0.05, ** *p* < 0.01, and *** *p* < 0.001 versus the vehicle group.

To determine whether RAM’s inhibition of intracellular ROS generation was related to the antioxidant system, the expression patterns of antioxidant enzyme-related proteins (Nrf2, HO-1, catalase, GPx, and SOD2) were investigated in HT22 cells. Western blotting showed that the Nrf2 pathway was activated in a time-dependent manner when treated with RAM (200 µg/mL) in the presence of Aβ (10 µM). The level of Nrf2 protein was most strongly upregulated (by 50–60% compared with the control) by 2R-AM at 30 min after roasting (Figure 5). Catalase, HO-1, SOD2, and GPx were significantly upregulated, particularly by 2R-AM. Compared with the control, Catalase and HO-1 expression increased by approximately 80–100% and 70–90%, respectively. SOD2 and GPx expression increased by 60–80% at 30 min, indicating activation of antioxidant defenses by 2R-AM.

### 3.6. Regulation of Apoptosis

**Figure 6:**
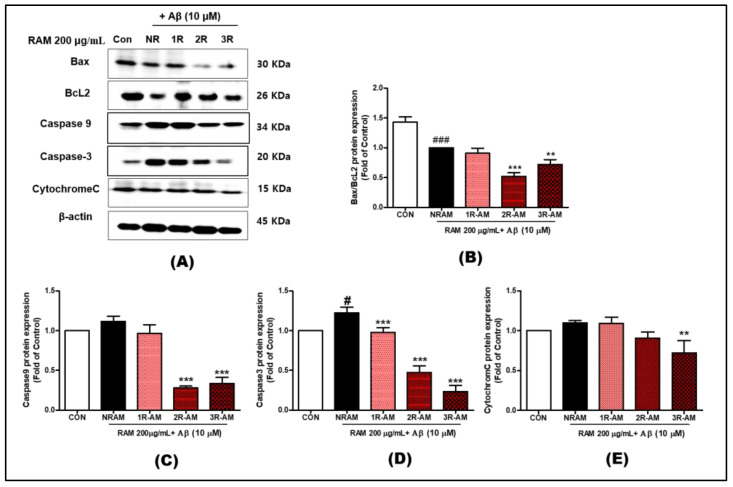
Representative Western blot analysis of Bax, Bcl-2, cytochrome c, caspase-9, and caspase-3 in Aβ-stimulated HT22 cells treated with RAM. (**A**) Western blot image showing Bax, Bcl-2, and β-actin, with β-actin as the loading control. (**B**) Ratio of Bax to Bcl-2 determined by Western blotting. (**C**–**E**) Western blot analysis shows RAM’s effect on cytochrome c, caspase-9, and caspase-3 levels in Aβ-treated HT22 cells, with β-actin used as the internal control. Values are presented as standard deviations. Statistical analyses were conducted by one-way ANOVA followed by Fisher’s LSD test. ^#^ *p* < 0.05, ^###^ *p* < 0.001 versus the control group. ** *p* < 0.01, and *** *p* < 0.001 versus the vehicle group.

To determine whether RAM can modulate apoptosis induced by Aβ toxicity, we examined the levels of endogenous apoptotic proteins (e.g., Bax) and anti-apoptotic proteins (e.g., Bcl-2) in HT22 cells (Figure 6A). RAM treatment decreased Bax levels in a time-dependent manner; 2R-AM reduced its level by 40–50%. Although RAM treatment significantly increased Bcl-2 levels, 2R-AM decreased its level by 40–50%, suggesting a shift towards apoptotic signaling (Figure 6B). RAM treatment increased the activation of caspase-9 and -3 while decreasing the levels of their cleaved forms by approximately 60% and 70%, respectively, after 30–40 min (Figure 6C–E). Additionally, the release of cytochrome c, a critical event in mitochondrial apoptosis, was reduced by approximately 50%, confirming apoptosis induction via the intrinsic pathway.

### 3.7. Neuroprotective Activity

**Figure 7:**
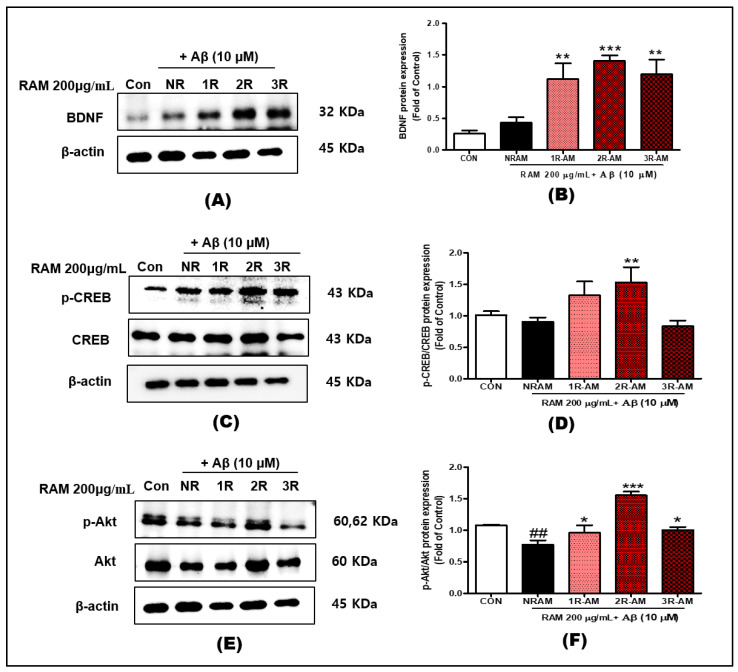
Representative Western blot image of p-Akt, Akt, p-CREB, CREB, BDNF, and β-actin in HT22 cells. β-actin was used as the internal loading control. (**A**) The ratio of BDNF to β-actin from Western blotting. (**B**) The ratio of BDNF to β-actin. (**C**) The ratio of p-CREB to CREB from Western blotting. (**D**) The ratio of p-CREB to CREB. (**E**) The ratio of p-Akt to Akt from Western blotting. (**F**) Ratio of p-Akt to Akt. β-actin was used as the internal loading control. Control groups were treated with the same volume of conditioned medium (0.2% DMSO). Values are presented as means ± standard deviations. Statistical significance was determined by one-way ANOVA followed by Fisher’s LSD test. ^##^ *p* < 0.01 vs. control group; * *p* < 0.05, ** *p* < 0.01, *** *p* < 0.001 vs. vehicle group.

We assessed the effect of RAM on the BDNF-Akt-CREB signaling pathway under oxidative stress. We investigated the expression of neurotrophic factor-related proteins, such as Akt, CREB, and BDNF (Figure 7). RAM treatment resulted in the activation of neuroprotective pathways, as evidenced by the upregulation of BDNF and phosphorylated CREB/p-CREB. 2R-AM increased BDNF expression by 20% (Figure 7B) and p-CREB expression by 50–70% (Figure 7D). Additionally, 2R-AM significantly increased the level of phosphorylated Akt/p-Akt, an essential mediator of cell survival, by 70–90% (Figure 7F). These findings indicated activation of the Akt signaling pathway, thereby regulating neurotrophic factors and inhibiting the neuronal apoptosis induced by oxidative stress.

### 3.8. Regulation of MAPK Signaling

**Figure 8:**
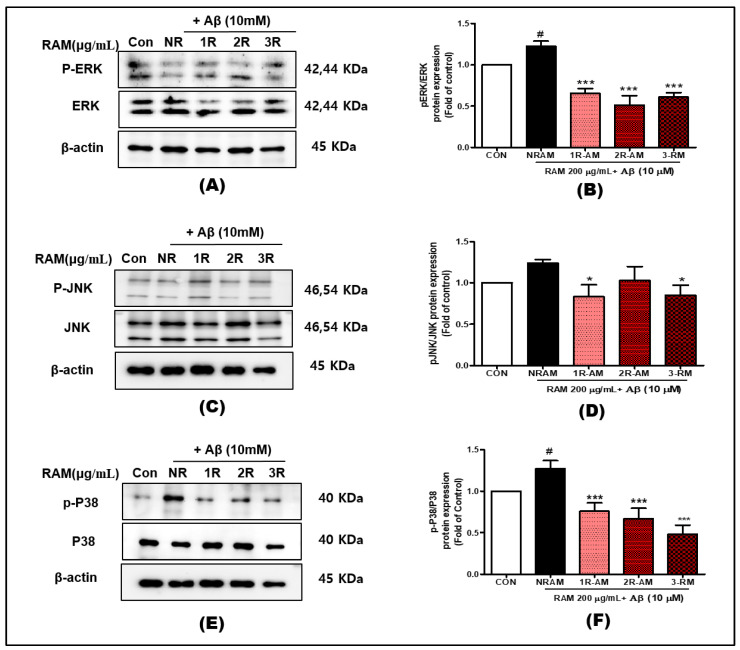
Representative Western blot image of p-ERK, ERK, p-JNK, JNK, p-P38, P38, and β-actin in HT22 cells. β-actin was used as the internal loading control. (**A**) The ratio of p-ERK to ERK was determined by Western blotting. (**B**) Ratio of p-ERK to ERK. (**C**) The ratio of p-JNK to JNK was determined by Western blotting. (**D**) The ratio of p-JNK to JNK. (**E**) The ratio of p-P38 to P38 was determined by Western blotting. (**F**) The ratio of p-P38 to P38. Control groups were treated with the same volume of conditioned medium (0.2% DMSO). Values are presented as standard deviations. Statistical analyses were conducted by one-way ANOVA followed by Fisher’s LSD test. ^#^ *p* < 0.05, versus the control group. * *p* < 0.05, and *** *p* < 0.001 versus the vehicle group.

MAPKs are involved in the oxidative stress response and the regulation of BDNF [28]. They transmit external stimuli intracellularly to promote proliferation and differentiation. RAM treatment increased the phosphorylated ERK (p-ERK) level to a peak of 30% at 20 min (Figure 8B). The phosphorylation of JNK was unaffected by RAM (Figure 8D), suggesting minimal involvement of this pathway. However, RAM increased the phosphorylated P38 (p-P38) level by 20% (Figure 8F), indicating activation of stress-related signaling pathways.

## 4. Discussion

Natural products have the property of regulating multiple targets, so they may be suitable for treating AD with complex pathological mechanisms This study focused on *Astragalus membranaceus* (AM), which has various physiological active effects among these natural products. It sought to explore the possibility of preventing and treating AD by increasing the physiologically active components of AM through the roasting process. Roasting is a processing method that uses heat to change plant components, enhancing antioxidant and anti-inflammatory effects. During this process, the concentration of neuroprotective components such as isoflavones may increase [29]. The increased antioxidant activity of heat-treated AM extracts is due to temperatures above 200 °C, which causes microscopic cracks and browning in the tissue, inducing chemical changes such as the Maillard reaction, thereby increasing antioxidant activity [29,30]. Dewanto et al. reported that heat treatment destroys the cell wall and decomposes insoluble phenolic compounds, releasing bound phenolic acids during extraction [31]. This is similar to a study on the effect of heat treatment on the morphology of *Cassia tora* [32]. However, excessive roasting can destroy nutrients and impart bitterness. Accurate control of roasting time, temperature, and ethanol concentration is essential to optimize food composition [33]

In this study, the isoflavone content increased approximately 2-fold after roasting at 260 °C for 30 min, and no harmful substances such as benzopyrene were detected, confirming the safety of the process (Figure 2). This study is of great value because it standardized the manufacturing process of roasted AM according to composition (S1).

Our study focused on roasting AM to enhance its physiological activity and analyze its neuroprotective effects in neurodegenerative diseases. Roasting significantly increased the concentration of flavonoids, which are the main components of AM, especially isoflavone components such as calycosin and formononetin. R-AM2 roasted for 30 min showed the most prominent physiological activity effect (Table 1), closely related to the decrease in ROS production (Figure 4), the increase in antioxidant protein expression, and the inhibition of apoptotic factors such as Bax and caspase-3. In particular, the Bax level decreased by approximately 40–50%, while the Bcl-2 level increased at the same rate, indicating an apparent inhibition of apoptosis (Figure 6). In addition, the activation of caspase-9 and caspase-3 was reduced, and cytochrome C release was inhibited, confirming that apoptosis via the mitochondrial pathway was inhibited. These results demonstrate that increased Bcl-2 and decreased Bax expression effectively suppressed oxidative stress and cell death in HT22 neurons induced by Aβ accumulation, suggesting that this balance regulation plays a vital role in neuroprotection.

This study confirmed that roasted AM exerted neuroprotective effects by activating the AKT/CREB/BDNF and Nrf2-mediated antioxidant pathways. In particular, AM roasted for 30 min (2R-AM) significantly increased the expression of antioxidant enzymes such as catalase, HO-1, SOD2, and GPx by activating the Nrf2 pathway (Figure 5). The upregulation of these enzymes played an essential role in protecting neurons by inhibiting ROS production and powerfully activating the antioxidant defense mechanism. A previous study reported that ginseng saponin’s neuroprotective effect was enhanced by increasing the level of trace ginsenosides through heat treatment. Nrf2-mediated antioxidant signaling was upregulated in this process, and MAPK-mediated apoptotic signaling was downregulated, protecting PC12 cells from glutamate-induced oxidative stress [34]. These results support the neuroprotective effect of roasted AM through a mechanism similar to this study [26].

Our results showed that RAM differentially regulates the MAPK signaling pathway. 2R-AM increased the level of p-ERK by approximately 30% and that of p-P38 by approximately 20%, suggesting activation of stress-related signaling (Figure 8). In contrast, its effect on the p-JNK level was minimal, indicating that the p-JNK pathway was not involved in the neuroprotective mechanism. The minimal change in the p-JNK level is likely because JNK mediates the response to pathological stimuli. In contrast, the AKT/CREB/BDNF pathway is implicated in neuroprotection and survival signaling [35]. The AKT/CREB/BDNF pathway is an important pathway that promotes neuronal survival and suppresses Aβ-induced toxicity. In particular, BDNF (brain-derived neurotrophic factor) plays an essential role in the growth and differentiation of neurons and reduces neurotoxicity due to Aβ accumulation [36]. Our study results showed that 2R-AM increased the expression of BDNF and p-CREB and increased the levels of p-Akt and p-CREB by 70–90%, promoting neuronal survival and enhancing resistance to oxidative stress (Figure 7). The increased expression of BDNF and p-CREB suggests that the neuroprotective effect of AM is mediated by the AKT/CREB/BDNF signaling pathway. In this study, the 20% increase in BDNF expression and 50–70% increase in p-CREB significantly enhanced neuronal survival and resistance to oxidative stress. This can be considered an important finding for the treatment of neurodegenerative diseases related to BDNF deficiency, especially Alzheimer’s disease. Nam et al. [37] reported that the BDNF/β-actin, p-ERK/ERK, and p-CREB/EREB ratios were significantly increased in mice injected with scopolamine and administered *Platycodon grandiflorum* root extract, consistent with our results. Therefore, the activation of AKT/CREB/BDNF signaling promotes the survival and growth of neurons. Consequently, the activation of ERK by this pathway promotes the differentiation and development of neurons, whereas Akt promotes neuronal survival.

However, this study has several limitations. First, the effect of RAM on cell death and the AKT/CREB/BDNF signaling pathway under controlled conditions is unknown. Additionally, further studies are required to determine whether other signaling pathways are involved in the protective effect of RAM against Aβ-induced toxicity. Second, the protective effect of RAM on hippocampal cells alone is insufficient to conclude a protective effect on the brain. For this reason, we are engaged in a follow-up study using an animal model of AD. In vivo experiments would have provided more substantial evidence of the therapeutic potential of RAM. Nevertheless, our findings suggest a novel therapeutic strategy for neurodegenerative diseases based on enhancing the pharmacological activity of AM. Although the antioxidant effects of the active ingredients of AM have been investigated, few studies have evaluated structural modifications of these active ingredients. Such studies may facilitate the development of novel neuroprotective agents. Therefore, the roasting-mediated enhancement of the physiological activities of AM components has important implications for the development of new neuroprotective agents. 

In conclusion, this study demonstrated that it is possible to enhance the physiological activity of *Astragalus membranaceus* (AM) through roasting and that this effectively protects neurons and promotes survival in neurodegenerative diseases. These results suggest the possibility of the development of a natural product-based neuroprotective agent.

## 5. Conclusions

RAM prevented Aβ-induced neurotoxicity in hippocampal cells by exerting antioxidant and anti-apoptotic effects. Roasted AM extract increased BDNF levels in HT22 cells, indicating prophylactic potential for neurodegenerative diseases associated with BDNF deficiency, such as dementia. This neuroprotective effect was primarily mediated by activation of the AKT/CREB/BDNF signaling pathway and inhibition of neuronal apoptosis, thus promoting neuronal survival. The findings suggest that roasted AM has therapeutic potential for degenerative brain diseases such as AD.

## 6. Patents

The following patent application has been made: Patent application name (10-2023-0185640): Method for manufacturing roasted *Astragalus membranaceus* with increased cognitive function improvement effect.

## Figures and Tables

**Table 1:** Isoflavone contents of roasted *Astragalus membranaceous* water extracts with different roasting times.

Samples	Calycosin 7-Glucoside	Ononin	Calycosin	Formononetin
**NR-AM (Con)**	708.04 ± 23.47	320.97 ± 13.04	229.51 ± 7.77	94.00 ± 11.99
**1R-AM (20 min)**	709.88 ± 11.85	497.55 ± 10.07	247.95 ± 4.81	104.18 ± 2.74
**2R-AM (30 min)**	570.43 ± 5.46	828.87 ± 12.61	307.69 ± 11.09	123.56 ± 8.75
**3R-AM (40 min)**	539.19 ± 27.01	716.30 ± 24.47	308.70 ± 2.09	113.86 ± 4.91

## Data Availability

Data is contained within the article.

## Abbreviations

Aβ	amyloid-beta
AD	Alzheimer’s disease
BBB	blood-brain barrier
BDNF	brain-derived neurotrophic factor
CNS	central nervous system
CREB	cAMP-responsive element binding protein
CAT	catalase
GPx	glutathione peroxidase
HO-1	heme oxygenase-1
HT22	hippocampal neuronal cells
MAPK	mitogen-activated protein kinase
Nrf2	nuclear factor erythroid 2 related factor 2
ROS	reactive oxygen species
SOD	superoxide dismutase
